# Chloroquine or Chloroquine-PI3K/Akt Pathway Inhibitor Combinations Strongly Promote γ-Irradiation-Induced Cell Death in Primary Stem-Like Glioma Cells

**DOI:** 10.1371/journal.pone.0047357

**Published:** 2012-10-16

**Authors:** Elke Firat, Astrid Weyerbrock, Simone Gaedicke, Anca-Ligia Grosu, Gabriele Niedermann

**Affiliations:** 1 Department of Radiation Oncology, University Hospital Freiburg, Freiburg, Germany; 2 Department of Neurosurgery, University Hospital Freiburg, Freiburg, Germany; University of Florida, United States of America

## Abstract

We asked whether inhibitors of the phosphatidylinositol 3-kinase (PI3K)/Akt pathway, which is highly active in cancer stem cells (CSCs) and upregulated in response to genotoxic treatments, promote γ-irradiationγIR)-induced cell death in highly radioresistant, patient-derived stem-like glioma cells (SLGCs). Surprisingly, in most cases the inhibitors did not promote γIR-induced cell death. In contrast, the strongly cytostatic Ly294002 and PI-103 even tended to reduce it. Since autophagy was induced we examined whether addition of the clinically applicable autophagy inhibitor chloroquine (CQ) would trigger cell death in SLGCs. Triple therapy with CQ at doses as low as 5 to 10 µM indeed caused strong apoptosis. At slightly higher doses, CQ alone strongly promoted γIR-induced apoptosis in all SLGC lines examined. The strong apoptosis in combinations with CQ was invariably associated with strong accumulation of the autophagosomal marker LC3-II, indicating inhibition of late autophagy. Thus, autophagy-promoting effects of PI3K/Akt pathway inhibitors apparently hinder cell death induction in γ-irradiated SLGCs. However, as we show here for the first time, the late autophagy inhibitor CQ strongly promotes γIR-induced cell death in highly radioresistant CSCs, and triple combinations of CQ, γIR and a PI3K/Akt pathway inhibitor permit reduction of the CQ dose required to trigger cell death.

## Introduction

Glioblastoma multiforme (GBM) WHO grade IV is the most common and the most aggressive brain tumor. It is uniformly fatal, and standard treatment with surgical resection plus temozolomide-based radiochemotherapy gives a median survival of only 14.6 months [1]. Highly therapy-resistant tumor stem cells appear to be at least partly responsible for the limited efficacy of current therapies [2], [3]. Phosphatidylinositol-3-kinase (PI3K)/Akt (protein kinase B) signaling is aberrantly activated in glioblastomas and other tumors, often due to mutation or loss of the Phosphatase and Tensin homolog (PTEN) antagonizing class I PI3K signaling, or to amplification or overexpression of growth factor receptors acting upstream of class I PI3K [4], [5]. Constitutive activation of PI3K/Akt signaling is associated not only with aggressive tumor growth but also with resistance to radio- and chemotherapy. Upregulation of the PI3K/Akt pathway in response to genotoxic treatments contributes to this resistance [6]. PI3K/Akt signaling stimulates a large variety of downstream molecules, some through the mammalian target of rapamycin (mTOR). Both autophagy, a lysosome-dependent degradation and recycling pathway triggered primarily as a survival response to various sublethal stresses, and apoptosis, the most common form of programmed cell death, are regulated by PI3K/Akt/mTOR signaling [7]–[9]. The PI3K/Akt pathway is also regarded as a stemness pathway important for survival of cancer stem cells (CSCs) [10].

GBM apparently conforms to the CSC model, being one of the best-characterized solid tumors studied under this aspect [2], [3]. According to the CSC model, many tumors may be driven by a subpopulation of stem-like cells, often termed CSCs. CSCs in general and stem-like glioma cells (SLGCs) in particular have been shown to be extraordinarily resistant to γ-irradiation (γIR) and chemotherapeutics. Blocked apoptosis and induction of autophagy appear crucial for this resistance contributing to the survival of genotoxically treated SLGCs [3], [11].

Contradictory findings on the effects of combinations of γIR with PI3K/Akt pathway inhibitors have been obtained for conventional glioma cell lines. Both lack of sensitization and strong sensitization to γIR-induced cell death have been reported [12]–[17]. Two studies have described sensitization of stem-like tumor cells to γIR by the Akt inhibitor perifosine in a transgenic mammary and in a medulloblastoma mouse tumor model [18], [19]. However, almost nothing is known about the effects of PI3K/Akt pathway inhibitors on the radiosensitivity of human patient-derived CSCs. Only one study reported enhanced caspase activity in a γ-irradiated SLGC line pretreated with the PI3K inhibitor LY294002 or a cytotoxic dose of Akt inhibitor III (SH6) [20].

The initial aim of the present study was to examine whether various types of pharmacological PI3K/Akt pathway inhibitors promote γIR-induced cell death when given to a panel of highly radioresistant primary SLGC lines. As the inhibitors did not generally enhance cell death but autophagy was observed, we also examined triple combinations with chloroquine (CQ), a clinically applicable autophagy inhibitor known to trigger apoptosis in conventional autophagic tumor cells. We show here for the first time that triple combinations of γIR with CQ and selected PI3K/Akt pathway inhibitors are strongly cytotoxic for highly radioresistant CSCs at low doses of CQ and that CQ alone, at slightly higher doses, strongly promotes γIR-induced cell death in highly radioresistant CSCs. Strong cell death observed in double and triple combinations with CQ occurred through apoptosis triggered by inhibition of late autophagy.

## Results

### Akt status and inhibition of Akt by PI3K/Akt pathway inhibitors

**Figure 1:**
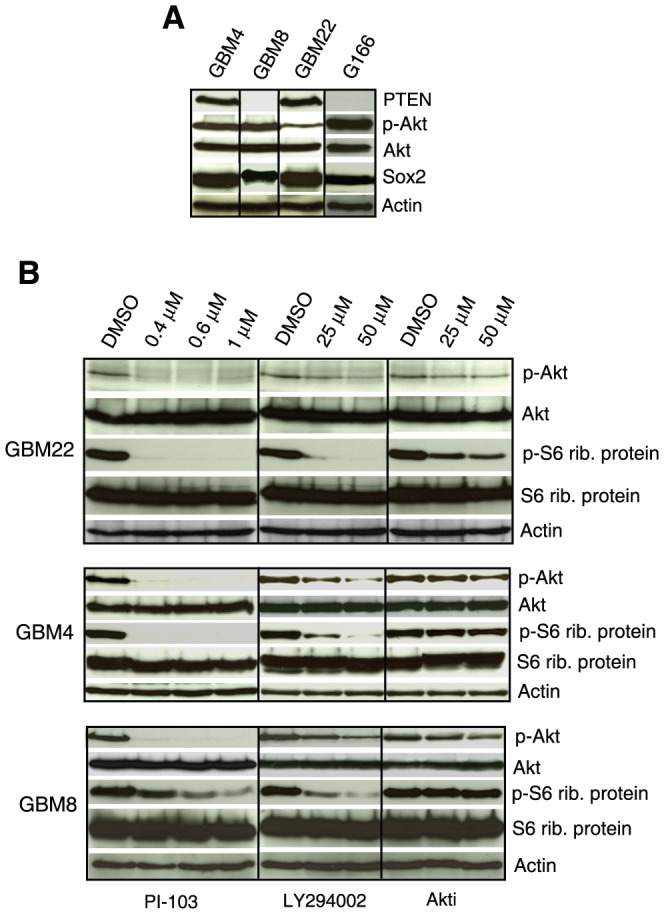
PI3K/Akt pathway activation status and inhibition of Akt phosphorylation by PI3K/Akt pathway inhibitors in primary SLGCs. (**A**) Western blot analysis of basal Akt, p-Akt, and PTEN expression levels. Sox2 is shown as a stemness marker and β-Actin as a loading control. (**B**) Inhibition of phosphorylation of Akt (serine 473) and of S6 ribosomal protein by Akt inhibitor III (Akti), LY294002 and PI-103. The cells were treated with the inhibitors for 2 h before collecting the samples for analysis.

We examined short-term SLGC lines from four different GBMs (GBM4, 8, 22 and G166). The lines express neural stem- and progenitor markers, differentiate upon exposure to FBS or retinoic acid, are tumorigenic upon serial xenotransplantation, and are highly resistant to γIR [21], [22]. All four lines showed expression of activated Akt (pAkt) phosphorylated at serine 473. Two of the three lines with high pAkt levels did not express PTEN, which negatively regulates Akt activity (Fig. 1A).

We next determined for these lines the inhibitory capacity of three different PI3K/Akt pathway inhibitors: the broad-range PI3K inhibitor LY294002, the phosphatidylinositol ether lipid analog Akt inhibitor III (SH-6), and PI-103, a pyridinylfuranopyrimidine compound, being a dual inhibitor of class I PI3K and mTOR [23] (Fig. 1B). Submicromolar doses of PI-103 completely inhibited phosphorylation of Akt (at serine 473) and of ribosomal protein S6, a major downstream target of the PI3K/Akt/mTOR pathway, which regulates protein synthesis and cellular proliferation. LY294002 also inhibited the phosphorylation of these two proteins efficiently. Inhibition by Akt inhibitor III was weaker, particularly in lines with high expression of pAkt (Fig. 1B).

### PI3K/Akt pathway inhibitors do not generally promote IR-induced SLGC death

**Figure 2:**
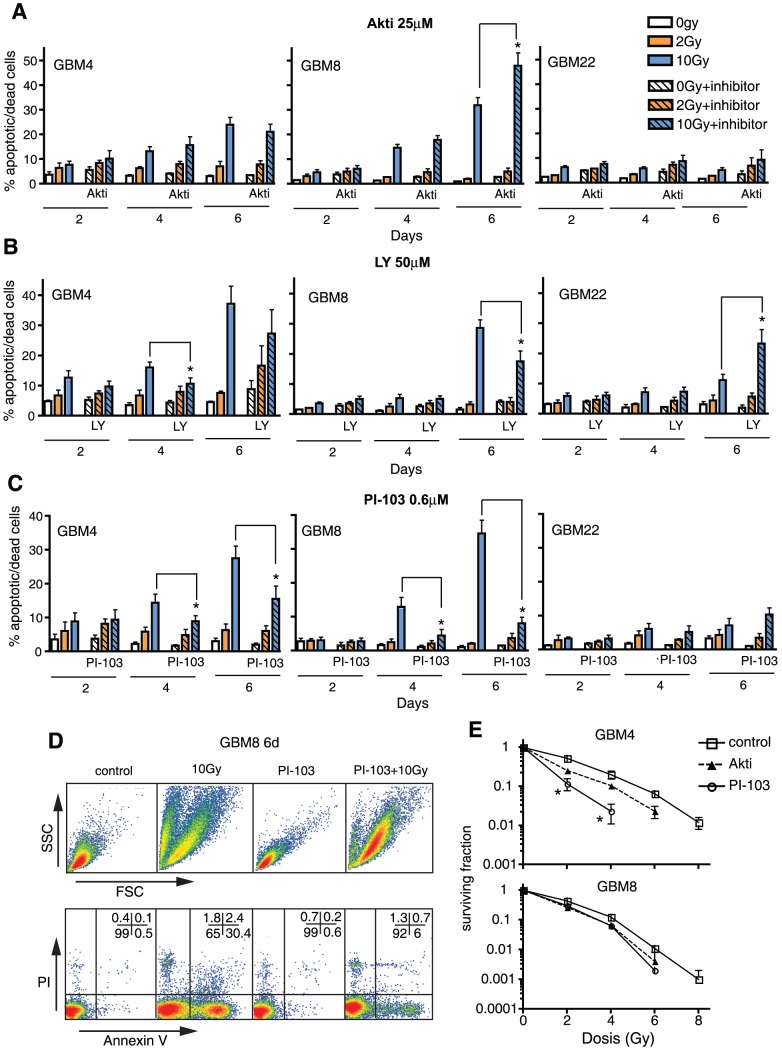
PI3K/Akt pathway inhibitors do not generally promote γIR-induced cell death in primary SLGCs. (**A**–C) Percentage of annexin V/PI positive cells 2, 4, or 6 d after irradiation with 0, 2, or 10 Gy. Prior to irradiation, the cultures were pretreated for 1 h with Akt inhibitor III, LY294002 or PI-103. (D) Example of flow cytometric cell death analyses. The population with decreased forward light scatter (FSC) in the 10 Gy-irradiated sample reflects cell shrinkage and fragmentation typical of apoptosis. Early and mid apoptotic cells are annexin V-positive but still exclude PI; late apoptotic cells with compromised membrane integrity are annexin V/PI-double positive. (**E**) Clonogenic survival 14 d after treatment with γIR +/− Akt inhibitor III (25 µM) or PI-103 (0.6 µM). Experiments were performed in triplicates. Data in A–C represent means ± SD of three independent experiments. Statistical significance is indicated by an asterisk (p<0.05).

Even though it only partially inhibited Akt phosphorylation, Akt inhibitor III reduced SLGC numbers strongly at 50 µM (Fig. S1A). The strong numerical reduction was associated with strong apoptosis. However, this strong apoptosis was not further promoted by additional γIR (Fig. S1B). In the GBM4 and GBM8 lines, which both have nonfunctional p53, we usually observe moderate apoptosis (associated with mitotic catastrophe) later than 4 days after applying moderate or high doses of γIR [21] (Fig. 2A–D). At the subtoxic concentration of 25 µM, Akt inhibitor III enhanced this delayed apoptosis slightly in GBM8 but not in the other SLGC lines.

50 µM LY294002 or 0.6 µM PI-103 strongly inhibited cellular proliferation (Fig. S1A) but did not generally induce apoptosis (based on annexin V exposure and forward/side scatter characteristics), nor did they generally promote radioinduced cell death (as assessed by propidium iodide [PI] uptake). Surprisingly, LY294002 and to a larger extent PI-103 even reduced apoptotic and dead cell numbers in γ-irradiated GBM4 and GBM8 SLGCs (Fig. 2B–D). Hence, the sensitizing effects observed in clonogenic assays, evaluating colony formation from single cells, probably mainly reflect reduced proliferation (Fig. 2E).

**Figure 3:**
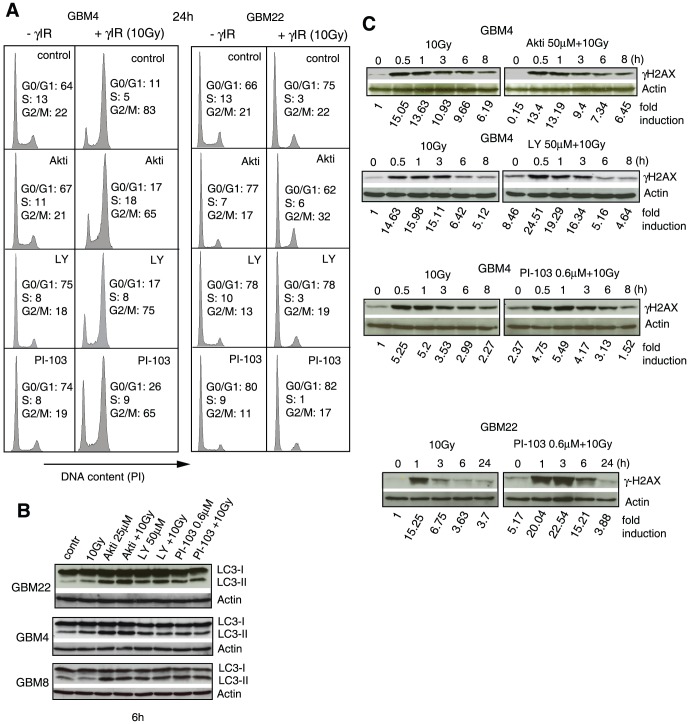
PI3K/Akt pathway inhibitors reduce G2M arrest and induce autophagy in γ-irradiated SLGCs. (**A**) Cells were treated with Akt inhibitor III (25 µM), LY294002 (50 µM), or PI-103 (0.6 µM) for 1 h and then irradiated with 10 Gy. The cell cycle analyses were performed 24 h later. (**B**) Conversion of cytosolic LC3-I to autophagosome-associated LC3-II, and (**C**) kinetics of H2AX-phosphorylation, a measure of DNA damage; the SLGCs were pretreated with PI3K/Akt pathway inhibitors for 1 h before irradiation with 10 Gy. Representative Western blots are shown.

When used as single agents, the reduced proliferation induced by all three inhibitors was associated with G0/G1 arrest (Fig. 3A). As expected, γIR caused G1 arrest in line GBM22 (the only line with functional p53 [21]) and G2M arrest in the lines with non-functional p53 (e.g., GBM4). γIR-induced G2M arrest in lines with non-functional p53 was reduced in the presence of the inhibitors, particularly PI-103. This likely contributes to reduced γIR-induced apoptosis, since we previously found a correlation among G2M arrest, mitotic catastrophe and delayed apoptosis in γ-irradiated p53-deficient SLGCs [21].

Since autophagy is usually upregulated as a survival mechanism in response to PI3K/Akt pathway inhibitors and in many cell types also in response to γIR [9], [24]–[27], we also examined whether autophagy is induced in SLGCs treated with γIR and/or the three different PI3K/Akt pathway inhibitors. As shown in Fig. 3B, a few hours after the treatment, conversion of microtubule-associated protein light chain 3 (LC3)-I into the lipidated LC3-II, which is incorporated into autophagic vesicles during autophagosome formation [28], could indeed be detected by Western blot. Autophagy induction could be confirmed by conducting autophagic flux assays with the late stage autophagy inhibitor Bafilomycin A1, which blocks lysosomal acidification by inhibiting the vacuolar proton pump, V-ATPase [29](Fig. S2).

LY294002 and, in one of two lines tested, PI-103 significantly enhanced radioinduced serine 139 phosphorylation of histone H2AX (Fig. 3C), a marker for DNA double-strand breaks. This is consistent with off-target inhibition of DNA-dependent protein kinase (DNA-PK) by both inhibitors [23]. The much stronger PI-103-mediated γH2AX-increase in GBM22 compared to GBM4 presumably reflects the major role of DNA-PK in non-homologous end-joining, the dominant DNA repair mechanism in G1 [30], where most of the GBM22 SLGCs arrest upon γIR (see Fig. 3A). Accordingly, the DNA-PK inhibitor (NU7441) increased residual γH2AX more strongly in GBM22 than in GBM4 SLGCs (Fig. S3). However, the increase in DNA double-strand breaks in PI-103-treated GBM22 SLGCs seemed primarily associated with a strong proliferation block rather than with cell death during our observation period of up to 2 weeks (see below).

In established tumor cell lines, traditionally cultured with FBS, used as controls, Akt inhibitor III enhanced γIR-induced cell death/apoptosis relatively strongly in three of four lines examined. Ly294002 had either a positive, negative or no effect, while PI-103 also here tended to suppress γIR-induced cell death (Fig. S4).

### Promotion of IR-induced cell death by CQ-PI3K/Akt pathway inhibitor combinations or CQ alone

Since none of the three PI3K/Akt pathway inhibitors readily enhanced IR-induced cell death in our SLGC lines but autophagy was induced, we hypothesized that triple combinations with the clinically applicable autophagy inhibitor CQ might be effective. CQ, as a weak base, raises the lysosomal pH and thereby, like Bafilomycin A1, inhibits late steps of autophagy (the fusion of autophagosomes with lysosomes and subsequent degradation of the cargo). Inhibition of late autophagy is a trigger for apoptotic cell death, particularly in cells exposed to autophagy-inducing agents [9], [31], [32].

**Figure 4:**
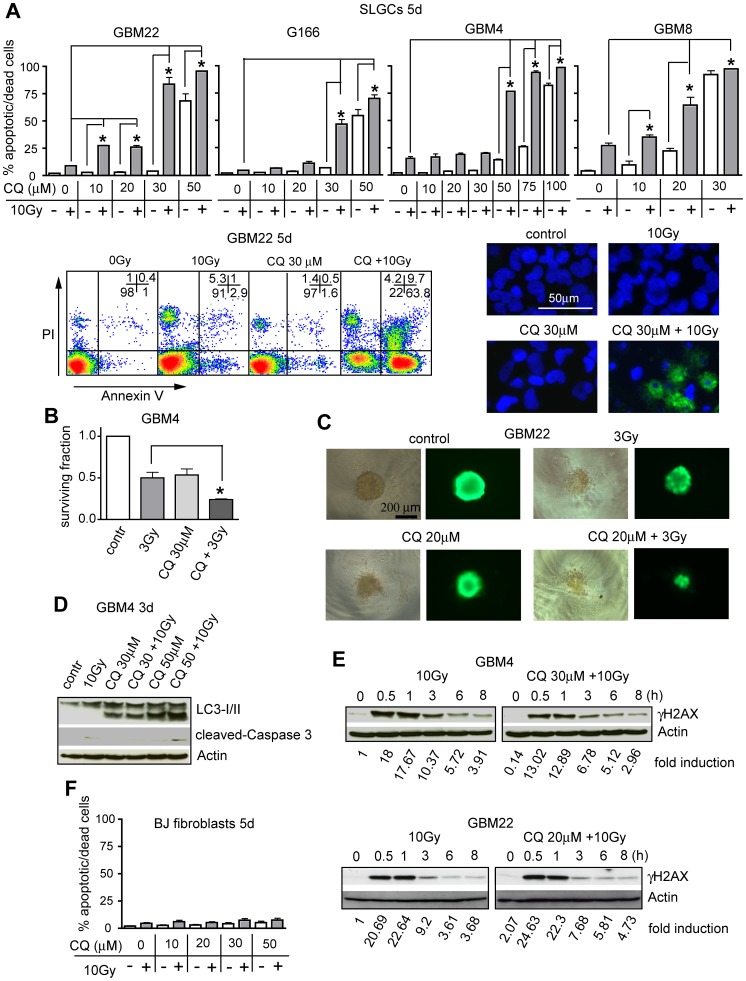
CQ and γIR synergistically induce strong apoptosis in SLGCs. (**A**) Upper panel: percentage of annexin V/PI positive cells 5 d after treatment with CQ alone (white bars) or CQ plus γIR (gray bars). The cells were pretreated with CQ for 1 h and then irradiated with 10 Gy. Lower left: example of the flow cytometric analysis of apoptotic and dead cells; lower right: apoptotic morphology observed after Hoechst 33342/annexin V staining at 3 d after combination treatment. (**B**) Reduced survival of clonogenic cells 13 d after combination treatment. (**C**) Size reduction of preformed neurospheres 7 d after combination treatment. Viability of neurospheres was determined after calcein-AM staining. (**D, E**) Representative Western blots showing the effect of CQ or CQ plus γIR on expression levels of activated caspase-3 and LC3-I/II conversion (**D**), as well as on phosphorylation of H2AX (γH2AX) (**E**). (**F**) Lack of cell death induction in human fibroblasts. In A and F, data represent means of three independent experiments. Experiments in B and C were performed twice in triplicates. Statistical significance is indicated by an asterisk (p<0.05).

To determine subtoxic concentrations of CQ for triple combination experiments, we first used CQ as a single agent or with 10 Gy γIR. Alone, CQ reduced the proliferation of SLGCs dose-dependently (Fig. S5A) and induced cell death (via apoptosis) at high concentrations (open bars in Fig. 4A). Apoptosis was induced at 30–50 µM CQ, except in GBM4 SLGCs where 100 µM CQ was required. GBM8, the line most susceptible to radio- or CQ-induced apoptosis, is the only one of the four SLGC lines that expresses the proapoptotic Bcl-2 protein Noxa (see below), and this expression was induced by CQ (Fig. S5B).

**Figure 5:**
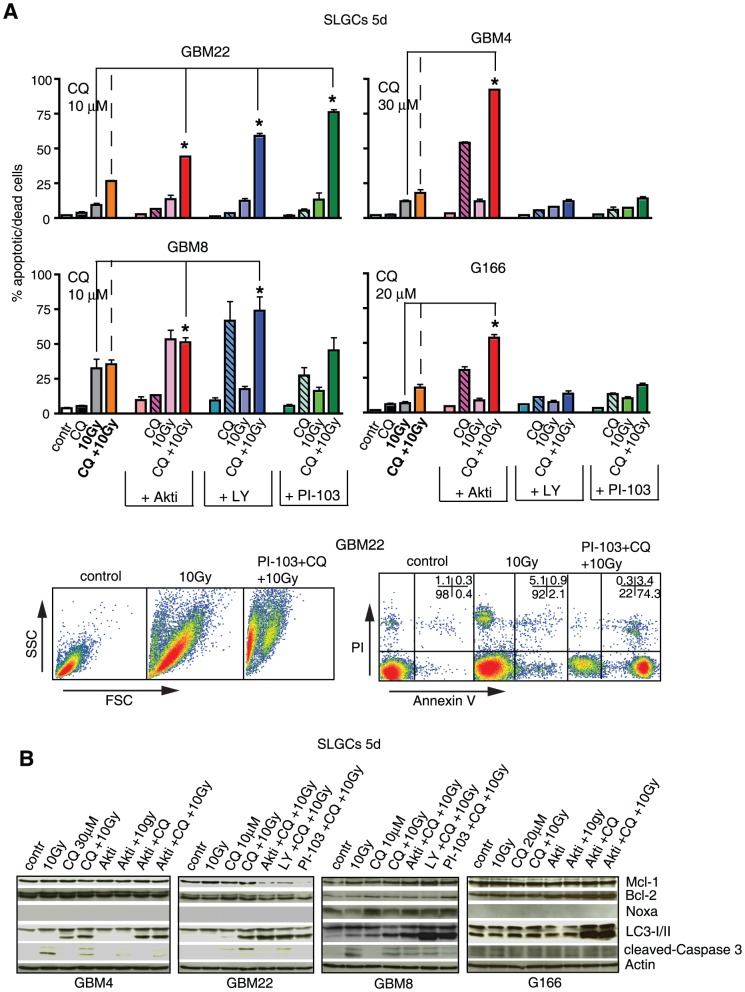
Triple combinations of γIR, a PI3K/Akt pathway inhibitor and low doses of CQ show additive to highly synergistic proapoptotic effects in primary SLGCs. (**A**) Percentage of annexin V/PI positive cells 5 d after treatment with 10 Gy, a PI3K/Akt pathway inhibitor (25 µM Akt inhibitor III, 50 µM LY294002, and 0.6 µM PI-103) and CQ (at the doses indicated), alone, or in double or triple combinations. Lower panel: example of flow cytometric analysis of GBM22 SLGCs showing that most cells treated with the triple combinations die by apoptosis. (**B**) Western blot analyses showing a strong accumulation of LC3-II in SLGC samples with high proportions of annexin V/PI positive cells. In addition, expression levels of pro- and antiapoptotic molecules and cleaved caspase-3 are shown. Data in (A) are means of three independent experiments. Statistical significance is indicated by an asterisk (p<0.05).

Testing the double combination of CQ and γIR, we found that CQ alone was already sufficient to strongly sensitize all four SLGC lines to radioinduced apoptosis (gray bars in Fig. 4A). For G166 and GBM4 SLGCs, this strongly enhanced apoptosis was seen at rather high CQ concentrations (30 or 50 µM, respectively). In contrast, highly synergistic proapoptotic effects were already detected at doses of 10 or 20 µM CQ in GBM22 and GBM8 SLGCs. Radiosensitization by CQ was also detected in clonogenic assays, as well as after treatment of preformed SLGC spheroids of defined size (Fig. 4B and C). Apoptosis induced by CQ alone or in combination with γIR was not accompanied by cell cycle alterations (Fig. S5C) and appeared to be largely caspase-independent (Fig. 4D). Levels of radioinduced DNA damage were unchanged or reduced in the presence of CQ (Fig. 4E), despite its highly synergistic effect with γIR. The highly synergistic proapoptotic effect was accompanied by massive accumulation of the autophagic marker LC3-II (Fig. 4D). As expected, CQ alone dose-dependently caused accumulation of LC3-II. However, the degree of LC3-I/II conversion was invariably higher in cultures treated with γIR plus CQ (Fig. 4D and see below). As the difference in LC3-II levels in the absence vs. presence of a late autophagy inhibitor reflects the number of autophagic vesicles that are transported to lysosomes [29], [33], this experiment confirms that autophagy is induced in the SLGCs in response to γIR. Moreover, it indicates that blocking the late steps of autophagy combined with enhanced autophagy induction causes massive accumulation of autophagosomes, a known trigger of apoptosis [31], [32], [34]. Note that after longer periods of single treatment with γIR (or PI3K/Akt pathway inhibitor) LC3-II levels were not enhanced anymore (see also Figs. 5B and S2 and compare to an earlier time point after treatment as shown in Fig. 3B). Since LC3-II itself is an autophagy substrate, this is most likely due to enhanced lysosomal delivery of LC3-II when autophagic flux is fully activated [29], [33]. CQ doses up to 50 µM did not sensitize normal human fibroblasts to radioinduced cell death (Fig. 4F), indicating some tumor specificity.

We next tested triple combinations of PI3K/Akt pathway inhibitors with 10 Gy γIR and CQ at CQ doses lower than those acting synergistically in the double combinations with γIR. Such triple combinations were indeed highly effective in all SLGC lines (Fig. 5A and Fig. S6). The strongest potentiation of radioinduced cell death was seen in SLGCs from GBM22. In this line, highly synergistic effects were achieved by combining a 10 Gy irradiation with a low dose of CQ (10 µM) and subtoxic doses of the three different PI3K/Akt pathway inhibitors. In GBM4 and G166 SLGCs, only Akt inhibitor III caused synergistic proapoptotic effects, and in GBM8 SLGCs, in which γIR alone caused some apoptosis, the effects were sub-additive to synergistic. The strong apoptosis induced by the triple combinations was accompanied by strong accumulation of the autophagic marker LC3-II. In GBM22 SLGCs, apoptosis was also accompanied by downregulation of the anti-apoptotic protein Mcl-1 (Fig. 5B).

### Effects of triple combination treatment at low doses of γIR, CQ, and PI-103

**Figure 6:**
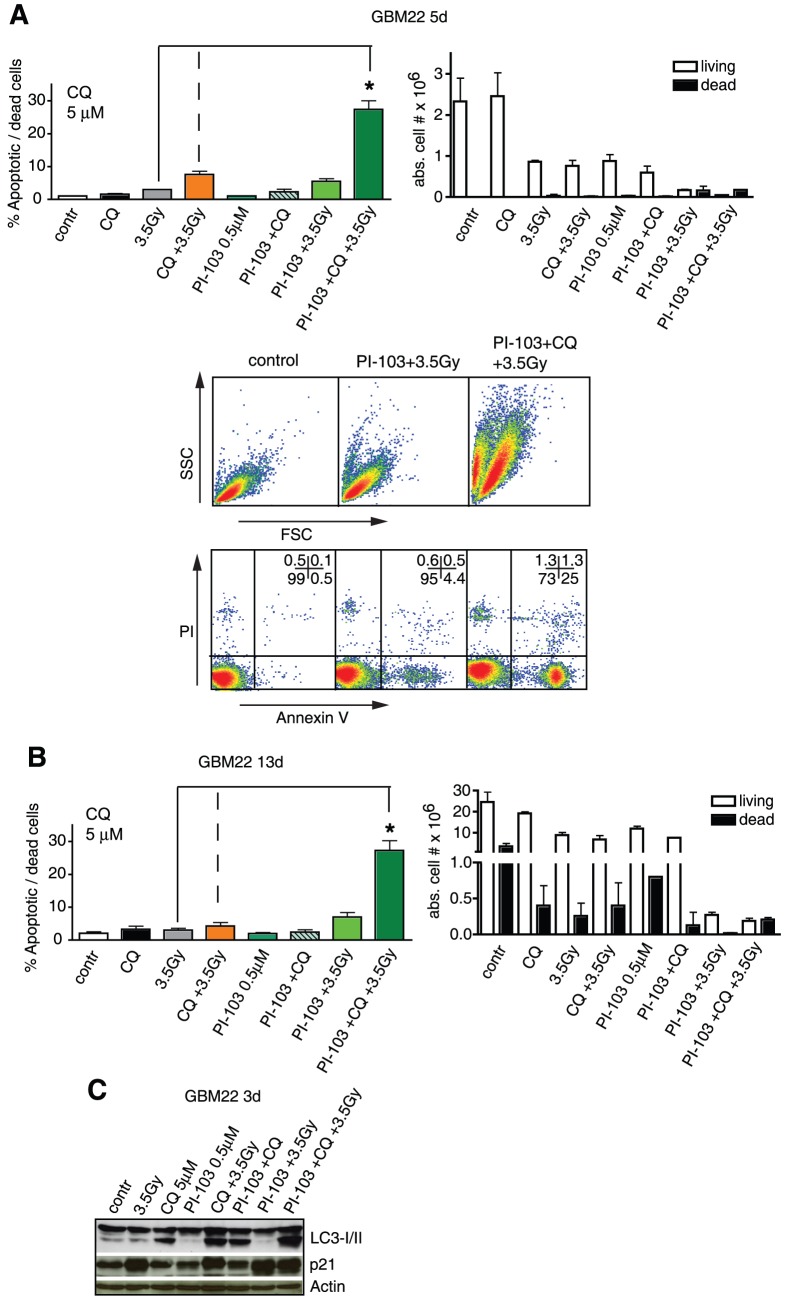
Low doses of γIR, CQ and PI-103 synergistically induce apoptosis in GBM22 SLGCs. (**A, B**) Left: Percentage of annexin V/PI positive cells 5 or 13 d after treatment with either γIR (3.5 Gy), CQ (5 µM) or PI-103 (0.5 µM) alone, with double or triple combinations. Right: cell numbers after trypan blue staining. Lower panel in A: flow cytometry data showing that cell death occurred primarily through apoptosis. (**C**) Western blot analysis of LC3-I/II conversion and of p21 expression levels.

Finally, we show that triple combinations of γIR, the dual class I PI3K/mTOR inhibitor PI-103, and CQ also caused synergistic proapoptotic effects when GBM22 SLGCs, which are completely cell death resistant at 10 Gy single dose irradiation, were treated with relatively low doses of all three components (3.5 Gy γIR, 0.5 µM PI-103, and 5 µM CQ), a result also of interest for a potential clinical application since lower doses cause fewer side effects. Although the double combination of PI-103 and γIR was strongly cytostatic (Fig. 6A and B right panels), significantly increased numbers of apoptotic or dead cells were only found in cultures treated with the triple combination including CQ. This was true for all time points tested up to d13 after the treatment (Fig. 6A and B). Also here, cell death/apoptosis was associated with the strongest conversion of LC3-I into LC3-II (Fig. 6C). This stronger LC3-I/II conversion in samples treated with the triple combination compared to double combinations with CQ indicates that double combinations of γIR and a PI3K/Akt pathway inhibitor more strongly activate autophagy than γIR or PI3K/Akt pathway inhibitor alone, and that more autophagic vesicles accumulate upon treatment with the triple combination compared to the double combinations with CQ [29], [33]. These results are similar to those obtained with bafilomycin A1 (see Fig. S2).

**Figure 7:**
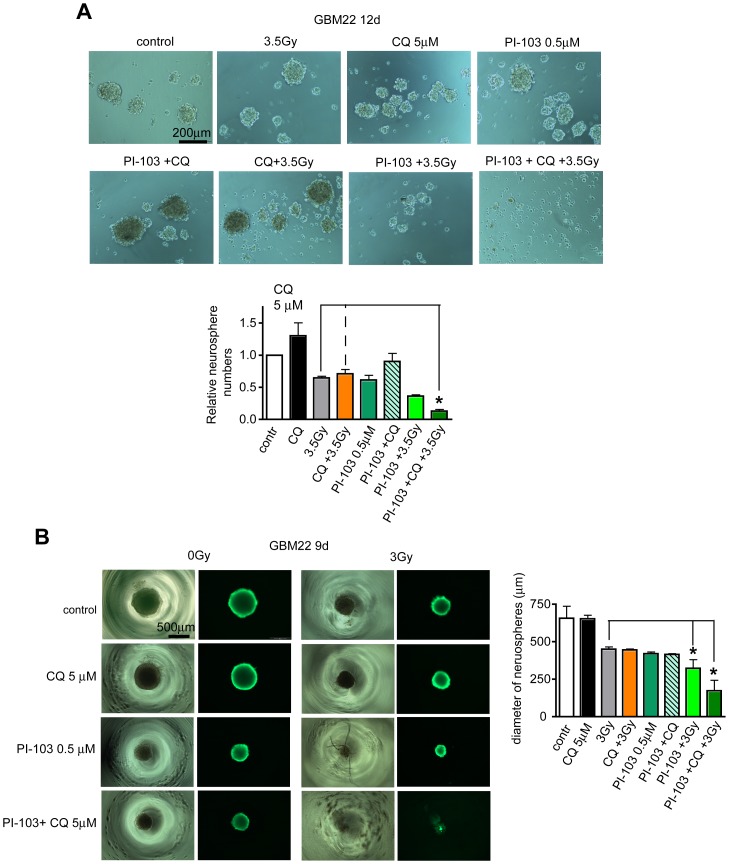
Synergistic effects of a low dose triple combination including PI-103 in stem cell surrogate assays. (**A**) Adherent monolayer cultures of GBM22 SLGCs were treated for 5 d as described in Fig. 6, then plated in serum-free medium to form neurospheres. Seven days later total neurospheres were counted and photographed. (**B**) Treatment of preformed 3D neurospheres. Cells were plated to form one neurosphere per well and 2 d later treated as indicated. 7 d after treatment, neurospheres were stained with calcein-AM to determine the viability and diameter of the spheres. Data in (A and B) are means of three independent experiments. Statistical significance is indicated by an asterisk (p<0.05).

We not only observed synergistic effects in apoptosis/global cell death assays, strong effects were also detected in sphere forming assays conducted as a stem cell surrogate assay to assess the effect on clonogenic cells. Also here, the triple combination most effectively prevented sphere formation (Fig. 7A). However, also in this assay the double combination of PI-103 and a 3.5 Gy irradiation turned out to be quite effective, possibly due to the strong cytostatic effect on SLGCs (see Fig. 6A and B). The triple combination was also most effective when three-dimensional spheres of defined size were treated. There were almost no viable cells several days after treatment as assessed by live-cell staining with a fluorescent viability dye (Fig. 7B). In contrast, no significant cell death could be detected in normal human fibroblasts treated with this triple combination (Fig. S7), demonstrating that not only double combinations of γIR and CQ (see Fig. 4F) but also this triple combination treatment exhibits some tumor cell specificity.

## Discussion

It is generally assumed that CSCs crucially contribute to the resistance of malignant tumors to chemo- and radiotherapy and that the Akt pathway is particularly important for CSC survival. However, how pharmacological inhibitors of the PI3K/Akt pathway affect post-irradiation survival and cell death of primary human CSCs has not been reported so far. Glioblastomas are resistant to conventional therapies. Novel therapeutic options are therefore urgently needed. To identify SLGC death-promoting agents, we selected one inhibitor of Akt and two PI3K/mTOR inhibitors to study their effects on the radioresponse of primary SLGCs. The two PI3K/mTOR inhibitors showed very strong cytostatic responses but, surprisingly, did not readily promote cell death of SLGCs and actually even tended to reduce it. Akt inhibitior III only slightly promoted γIR-induced cell death in one of the SLGC lines examined. The response to these double combinations was associated with autophagy and we show here for the first time that the late autophagy inhibitor CQ alone or in combination with selected PI3K/Akt pathway inhibitors strongly promotes γIR-induced cell death of primary human CSCs. Our study should be of high clinical relevance not only because a clinically applicable late autophagy inhibitor was used, but also because all data presented have been obtained from patients' tumor-derived stem-like tumor cells, a population thought to be critical for tumor progression and treatment resistance that must be eradicated in order to achieve long-term relapse-free survival.

Primary (p53-dependent) apoptosis is not a preferred reaction of solid tumor cells to genotoxic treatments. However, in most SLGC lines with nonfunctional p53, we usually detect some apoptosis later than 4 days after γIR with moderate or high single doses (e.g., 5 or 10 Gy) [21] as used in hypofractionated clinical treatment regimens [35]. As these SLGC lines show pronounced G2M arrest associated with mitotic catastrophe, this is presumably secondary apoptosis following a lethal mitotic catastrophe. In contrast, SLGCs with functional p53 merely arrest in G1 and show no cell death for over 2 weeks post-irradiation. During this period, we also do not see cell death in the p53-deficient SLGC line G166, which, despite G2M arrest, does not exhibit features of mitotic catastrophe after γIR [21]. Thus, although there are differences in the radioresponse of CSCs, there is a great need for CSC radiosensitizers, particularly for highly chemo- and radio-resistant malignancies.

In triple combinations with γIR and a PI3K/Akt pathway inhibitor, CQ strongly promoted SLGC apoptosis at concentrations between 5–30 µM, and in double combination with γIR in the range of 10–50 µM. Our finding that CQ strongly promotes cell death in γ-irradiated SLGCs may contribute to explaining the results of a clinical study by Sotelo *et al*., who reported that CQ, added to a conventional therapeutic protocol (surgery plus radiochemotherapy) for glioblastoma, doubled the median overall survival compared with control groups [36]. Radiosensitization by CQ has already been reported for conventional tumor cell lines, mostly in conjunction with hyperthermia [37], [38], but radiosensitization of glioma cells or highly radioresistant CSCs, and such strong synergistic proapoptotic effects of γIR and CQ as reported here uniformly for a panel of primary SLGCs have not been described before.

Can concentrations of 5–50 µM CQ be achieved therapeutically? CQ is a cheap antimalarial drug, also being used for treating rheumatoid arthritis, lupus erythematosus and other connective tissue disorders [39]. The peak blood plasma concentrations of approximately 1–5 µM reported in the literature for CQ [40] are equal to or slightly below the concentrations found here to radiosensitize SLGCs. However, CQ crosses the blood-brain barrier. It also accumulates in certain tissues and organs to very high levels, and values 10–30 times higher than blood plasma concentrations have been described for the brain [41]. Therefore, CQ concentrations required for radiosensitization of CSCs may indeed be achievable by systemic administration at least for some tumor entities including brain tumors. Controlled local application of CQ may be an alternative delivery strategy in brain tumors.

Is a certain molecular background associated with CQ-mediated radiosensitization of SLGCs? Strong cell death enhancement was observed for all four SLGC lines, including G166 and GBM22, which for at least up to two weeks post-irradiation, do not show any signs of cell death even after high single doses of γIR such as 10 Gy [21]. However, apoptosis at lower CQ dose (10 or 20 µM) was only observed in irradiated GBM22 and GBM8 SLGCs. The GBM22 line is the only line expressing functional p53, and GBM8 the only line expressing the proapoptotic Bcl-2 family member Noxa. A positive influence of functional p53 and an influence of Bcl-2 family members such as Bax, Bak, and Bcl-xL have already been noticed when conventional tumor cell lines were exposed to CQ as a single agent [42]–[45]. Noxa was found here to be induced by CQ. Noxa has recently been shown to promote autophagic cell death upon expression of the oncogenic protein Ras [46]. Future experiments are required to find out whether Noxa and p53 also contribute to the CQ-mediated cell death enhancement in γ-irradiated CSCs.

Mechanistically, the strong proapoptotic synergism of the γIR/CQ-cotreatment likely results from the combination of induction of autophagy (by the genotoxic treatment) with strong inhibition of the late steps of autophagy (by CQ) [9], [31], [32]. Clinically, radiotherapy may have an advantage over chemotherapy because it is a local genotoxic treatment. Induction of DNA damage by CQ described by others [47] could also explain enhanced γIR-induced apoptosis. However, we observed either no change or reduced radioinduced DNA damage as assessed by γH2AX. Reduction of DNA damage is consistent with the view that CQ has antimutagenic properties [48], which may also contribute to its antitumoral effects.

With two exceptions (Akt inhibitor III in GBM8 and Ly294002 in GBM22 SLGCs), we found either no increase or even reduction of radiogenic SLGC death in the presence of PI3K/Akt pathway inhibitors. Reduced cell death was observed for p53-deficient SLGCs cotreated with the PI3K/mTOR inhibitors LY294002 or PI-103. As we previously showed that p53-deficiency, proliferation and mitotic catastrophe associated with G2M arrest correlate with γIR-induced SLGC apoptosis [21], strongly decreased proliferation and diminished G2M arrest likely contribute to the apoptosis-inhibitory effects of LY294002 and PI-103 in γ-irradiated SLGCs in addition to the induction of autophagy. Lack of apoptosis and largely cytostatic effects have also been observed upon monotherapy with dual PI3K/mTOR inhibitors in conventional glioma lines [4], [23], [27]. At first glance, this appears surprising given the comparably strong inhibition of the important apoptosis regulator Akt by such inhibitors; strong Akt inhibition by PI-103 (a PI3K/pan mTOR inhibitor) may be due to inhibition of mTORC2, the main cellular kinase phosphorylating serine 473 of Akt [4]. The strong G1 cytostatic response in conjunction with autophagy appears to facilitate cell survival in the presence of such inhibitors.

In cultures treated with lethal triple combinations, apoptosis was associated with very strong LC3-I/II conversion, reflecting the accumulation of large amounts of autophagic vesicles, known to precede apoptotic cell death when late autophagy is blocked [31], [32], [34]. In the case of our triple combinations, strong autophagosome accumulation is caused by induction of autophagy with two different autophagy inducers (γIR and PI3K/Akt pathway inhibitor) and simultaneous impairment of autophagosome clearance by the lysosomotropic CQ (see Figs. 5B, 6C and S2). Reduction of the apoptosis-inducing threshold dose of CQ in triple combinations compared to double combinations with CQ can therefore probably be explained largely by the fact that the extent of autophagosome accumulation depends on two manipulations, induction of autophagy on the one hand and blockage of late autophagy on the other [9], [31], [32], [34]. However, in two SLGC lines (GBM4 and G166), only Akt inhibitor III was proapoptotic in the triple combinations. In some cases, the double combination of PI3K/Akt pathway inhibitor and CQ already caused some apoptosis (see Fig. 5A, hatched bars) as previously described for conventional prostate and glioma lines [24], [25]. Only in the case of GBM22, which has relatively low basic pAkt expression, did we observe strong synergistic effects for the triple combinations with all three PI3K/Akt pathway inhibitors. PI-103 induced synergistic proapoptotic effects on these SLGCs even when administered together with only 5 µM CQ, which might easily be achieved therapeutically, and low dose γIR (3 or 3.5 Gy). The highly synergistic proapoptotic effect at low doses of all three components of the PI-103/γIR/CQ triple combination is remarkable because the GBM22 SLGC line is extraordinarily resistant to cell death even when irradiated with 10 Gy [21].

Taken together, the PI3K/Akt pathway inhibitors we examined did not generally promote γIR-inducedcell death of SLGCs in double combination with γIR, but CQ or combinations of CQ with selected PI3K/Akt pathway inhibitors strongly promoted γIR-induced cell death in otherwise highly radioresistant SLGCs. Our findings warrant further preclinical evaluation and suggest that sensitivity testing of primary SLGCs toward double combinations of γIR with CQ or triple combinations of γIR, CQ and different PI3K/Akt pathway inhibitors could predict the treatment response of patients. Such combinations also look promising as CSC radiosensitizers in other malignancies. Our results may therefore be of great interest, given the more than 20 ongoing oncology trials testing CQ or its derivative hydroxy-CQ as monotherapy or in combination with other agents (clinicaltrials.gov).

## Materials and Methods

### Tumor samples, cell culture, and reagents

The GBM4, GBM8, and GBM22 SLGC lines were established from tumor samples of patients diagnosed as classical primary GBMs, as previously described [21]. Informed written consent was obtained before surgery according to the protocols approved by the local ethics committee of the University of Freiburg (Approval ID: 349/08). After tumor dissociation, tumor single cell suspensions were cultured under stem cell conditions, i.e., they were allowed to form spheres on low attachment plates (Corning) in serum-free Neurobasal Medium (Gibco) supplemented with epidermal growth factor (EGF)/fibroblast growth factor-2 (FGF-2; 20 ng/ml each), B27, non-essential amino acids, penicillin/streptomycin, glutamax and heparin. The SLGC line G166 [22] was purchased from Biorep (Milan, Italy). For experiments, the cultures were expanded in plates coated with extracellular matrix proteins (mouse sarcoma-derived ECM, Sigma) [22]. The glioma lines U87, U251 and LN229, the colon carcinoma line HCT116 and BJ normal human fibroblasts were obtained from the American Type Culture Collection (ATCC) and cultured in DMEM (Gibco) supplemented with 10% FBS, penicillin/streptomycin, non-essential amino acids, and 2-mercaptoethanol. Akt inhibitor III (SH-6) and LY294002 were purchased from Calbiochem; PI-103 was from BioVision and NU7441 from Tocris Bioscience. Chloroquine and Bafilomycin A1 were purchased from Sigma.

### γIR

Irradiations were performed using a Gammacell 40 ^137^Cs laboratory irradiator.

### Flow cytometric apoptosis and cell death assay

Cell cultures seeded 24 h before were treated with a PI3K/Akt-inhibitor or CQ or combinations of both drugs and then irradiated 1 h later. At the indicated time points, the cells were harvested, washed, and stained with Annexin V and PI using an Annexin V-FITC Kit from Miltenyi Biotec. Apoptotic (annexin V+) and dead cells (PI+) were measured by flow cytometry on a Cytomics FC 500 instrument from Beckman Coulter.

### Microscopic analysis of cell death morphology

Cells were treated with CQ for 1 h and then irradiated with 10 Gy. Three days later, the cells were stained with Annexin V and Hoechst 33342 using an Apoptotic/Necrotic/Healthy cells detection kit from PromoKine. Cells were analyzed using an Olympus BX41 fluorescence microscope equipped with a digital camera CC-12 soft imaging system (U-CMAD3, Olympus).

### Cell cycle analyses

24 h after seeding, cells were treated with a PI3K/Akt inhibitor or CQ and irradiated 1 h later. 24 h after irradiation, the cells were fixed with 70% ethanol and stored overnight at −20°C. Cells were then washed and incubated with propidium iodide (50 µg/mL) and RNase (100 µg/mL) for 2 h at 4°C. After washing, the cells were analyzed for DNA content by flow cytometry.

### Cell Growth and Viability Assay

An aliquot of cell suspension was mixed with Trypan blue solution, and the numbers of live (trypan blue negative) and dead cells (trypan blue positive) were counted under a microscope.

### Western blot analyses

Cell lysates were prepared in RIPA lysis buffer supplemented with protease inhibitor cocktail (Roche) and phosphatase inhibitors NaF and Na_3_VO_4_ (Sigma). 40 µg of cell lysates was separated by SDS-PAGE and blotted onto nitrocellulose. The blots were probed with the indicated antibodies and developed by enhanced chemiluminescence (Amersham Biosciences). The following antibodies were used: PTEN, Akt, phospho-Akt (Ser473), S6 ribosomal protein, phospho-S6 ribosomal protein (Ser235/236), Mcl-1, LC3B, cleaved caspase-3, p21 and phospho-Ser139-histone H2AX (Cell Signaling); actin and Bcl-2 (Santa Cruz Biotechnology); Noxa (Enzo); Sox2 (R+D Systems); HRP-conjugated secondary antibodies (Dianova). Quantification of signals was performed using Image Quant TL (Amersham Bioscience).

### Clonogenic assay

6 h after seeding, cells were treated and irradiated. After 14 d, colonies were fixed and stained with 0.5% crystal violet. Colonies with more than 50 cells were counted. Experiments were performed in triplicate.

### Neurosphere assay

Cells were seeded for adherent growth and 24 h later treated as indicated. At day 5 after treatment, the cells were trypsinized and equal numbers of living cells were seeded to form neurospheres in 48 well plates for an additional 7 days.

### Treatment of preformed neurospheres

300 cells were plated to yield one neurosphere per 96 well (V-bottom microplates). After 2 days neurospheres all of similar size had formed, and were treated as indicated. Seven days later, neurospheres were stained for viability with 2 µM calcein-AM (Sigma) for 30 min at 37°C and analyzed under a fluorescence microscope.

### Statistical analyses

All data are presented as mean ± SD and analyzed by Student's t test, two-tailed, with unequal variance. P<0.05 was considered significant.

## Supporting Information

Figure S1 **Effect of PI3K/Akt pathway inhibitors on proliferation and radioinduced cell death of SLGCs.** (A) Cells were pretreated with Akt inhibitor III, LY294002 or PI-103 for 1 h before irradiation. At the indicated times, cells were counted after staining with trypan blue. (B) Percentage of apoptotic and dead (annexin V/PI-positive) cells 2 or 4 d after irradiation with or without co-treatment with 50 µM Akt inhibitor III. Right panel: expression levels of Mcl-1 and cleaved Caspase 3 detected by Western blotting. Data in A and B are means of 3 independent experiments. Statistical significance is indicated by an asterisk (p<0.05).
(EPS)

Figure S2 **Example of a classical “autophagic flux” assay with the late stage autophagy inhibitor Bafilomycin A1.** Cells were treated or not with PI-103 (0.6 µM) with or without irradiation (10 Gy) for 24 h and for the last 4 h bafilomycin A1 was added at a concentration of 100 nM. Samples were then analyzed for LC3-I/II expression levels by Western blotting. Although increased LC3-I/II conversion could not be detected anymore 24 h after a single treatment with γIR or PI-103 (compare the 6 h treatment in Fig. 3B), the differences in LC3-II levels between samples treated plus/minus the late stage autophagy inhibitor Bafilomycin A1 indicate that treatment with γIR or PI-103 and even stronger the combination thereof induces the formation of autophagosomes. That, at this time point, LC3-II levels are not increased any longer in the absence of Bafilomycin A1 is likely due to enhanced delivery of LC3-II to lysosomes (i.e., enhanced autophagic flux) when autophagy is fully activated.
(EPS)

Figure S3 **Effects of a specific DNA-PK inhibitor on H2AX phosphorylation.** The cells were pretreated for 1 h with the DNA-PK inhibitor NU7441 (1 µM) before IR.
(EPS)

Figure S4 **Enhancement of radioinduced apoptosis by Akt inhibitor III and LY294002 in conventional tumor cell lines.** Tumor cell lines with different molecular backgrounds (PTEN/p53 status) were pretreated with Akt inhibitor III (25 µM), LY294002 (50 µM) or PI-103 (0.6 µM) for 1 h and then irradiated with 10 Gy. Three days later, the cells were stained with Annexin V/PI for assessing apoptosis and total cell death by flow cytometry. Data are means of three independent experiments. Statistical significance is indicated by an asterisk (p<0.05).
(EPS)

Figure S5 **Effect of CQ on proliferation, Noxa expression and cell cycle distribution of primary SLGCs.** (A) SLGCs were treated with CQ for 1 h at the doses indicated and then irradiated with 10 Gy. At d5 cells were counted after trypan blue staining. One representative experiment is shown. (B) Cells were incubated with CQ as indicated and analyzed for Noxa expression by Western blotting. (C) Cells were treated with CQ (30 µM for GBM4 and 10 µM for GBM22) for 1 h and then irradiated with 10 Gy. Cell cycle analyses were performed 24 h later.
(EPS)

Figure S6 **Assays to prove completeness of cell death.** GBM22 SLGCs were either not treated or treated with a triple combination of 10 Gy γIR, 0.6 µM PI-103, and 10 µM CQ. 1, 3 and 5 d after treatment, total cell numbers were counted (lower left) and photos were taken of the cultures (upper right) as well as of DAPI-stained samples (lower right). In the treated cultures, cell numbers were strongly decreased compared to the numbers of seeded cells, and large numbers of fragmented cells and nuclei were photographically detected. Apoptotic nuclear fragmentation was also measured by flow cytometry after staining of fixed cells with PI (upper left). Note that the sub-G1 content at d5 (82%) is very similar to the fraction of annexin V/PI-positive cells (appr. 75%) found at d5 in cultures treated with the same triple combination and analyzed by annexin V/PI-staining (see Fig. 5A, green bars in the upper left panel). Moreover, the large numbers of fragmented cells and nuclei shown on the photographs correspond very well to the changes in the flow cytometric forward scatter (an estimate of cell size) shown in the lower left panel of Fig. 5A.
(TIF)

Figure S7 **Higher resistance of normal human fibroblasts to the triple combination of γIR, PI-103 and CQ.** Normal human fibroblasts were treated exactly like the GBM22 SLGCs in Fig. 6. Five and 13 d after the treatment, the proportion of annexin V/PI-positive cells was not significantly increased and only intact cells were photographically detected.
(TIF)
