# Supplementary figures and images for: Chloroquine or Chloroquine-PI3K/Akt Pathway Inhibitor Combinations Strongly Promote γ-Irradiation-Induced Cell Death in Primary Stem-Like Glioma Cells
Source: PLoS One. 2012 Oct 16;7(10):e47357. doi: 10.1371/journal.pone.0047357 (PMC3473017; doi:10.1371/journal.pone.0047357)

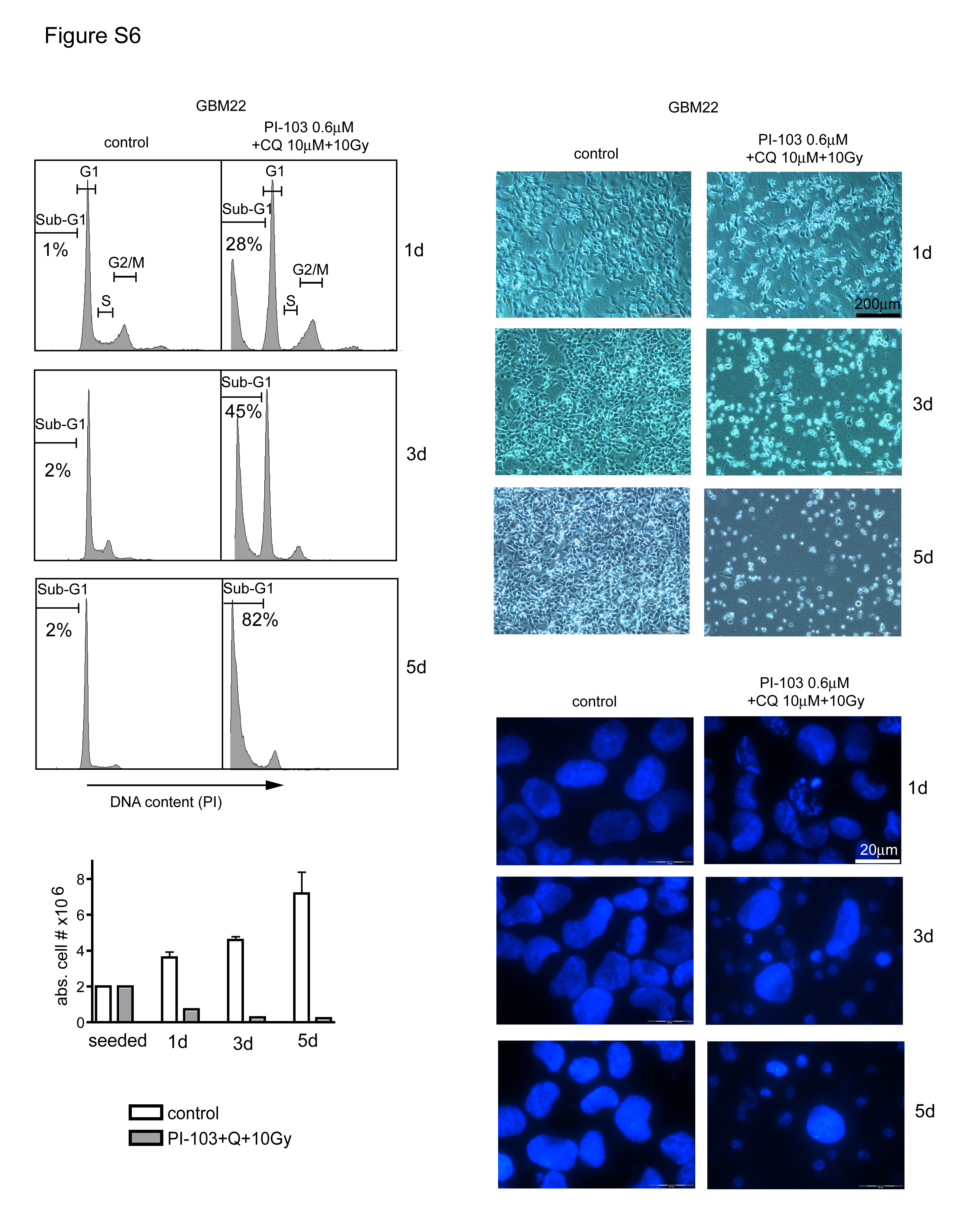

Supplement: Figure S6 — Assays to prove completeness of cell death. GBM22 SLGCs were either not treated or treated with a triple combination of 10 Gy γIR, 0.6 µM PI-103, and 10 µM CQ. 1, 3 and 5 d after treatment, total cell numbers were counted (lower left) and photos were taken of the cultures (upper right) as well as of DAPI-stained samples (lower right). In the treated cultures, cell numbers were strongly decreased compared to the numbers of seeded cells, and large numbers of fragmented cells and nuclei were photographically detected. Apoptotic nuclear fragmentation was also measured by flow cytometry after staining of fixed cells with PI (upper left). Note that the sub-G1 content at d5 (82%) is very similar to the fraction of annexin V/PI-positive cells (appr. 75%) found at d5 in cultures treated with the same triple combination and analyzed by annexin V/PI-staining (see Fig. 5A, green bars in the upper left panel). Moreover, the large numbers of fragmented cells and nuclei shown on the photographs correspond very well to the changes in the flow cytometric forward scatter (an estimate of cell size) shown in the lower left panel of Fig. 5A. (TIF) [file pone.0047357.s006.tif]

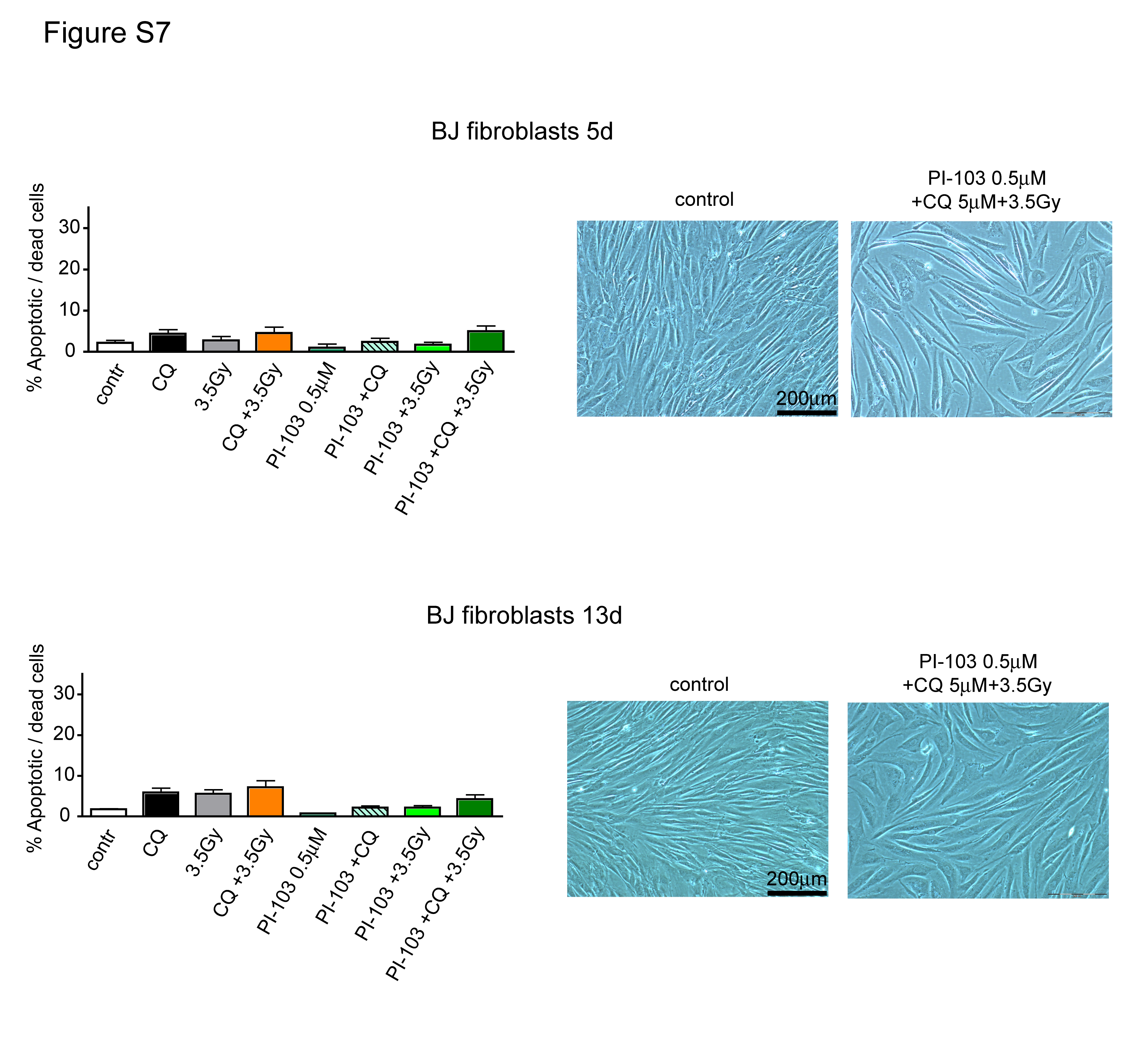

Supplement: Figure S7 — Higher resistance of normal human fibroblasts to the triple combination of γIR, PI-103 and CQ. Normal human fibroblasts were treated exactly like the GBM22 SLGCs in Fig. 6. Five and 13 d after the treatment, the proportion of annexin V/PI-positive cells was not significantly increased and only intact cells were photographically detected. (TIF) [file pone.0047357.s007.tif]
